# The Italian National Register of infants with congenital hypothyroidism: twenty years of surveillance and study of congenital hypothyroidism

**DOI:** 10.1186/1824-7288-35-2

**Published:** 2009-02-20

**Authors:** Antonella Olivieri, The Study Group for Congenital Hypothyroidism

**Affiliations:** 1 Dipartimento di Biologia Cellulare e Neuroscienze, Istituto Superiore di Sanità, Rome, Italy

## Abstract

All the Italian Centres in charge of screening, diagnosis, and follow-up of infants with congenital hypothyroidism participate in the Italian National Registry of affected infants, which performs the nationwide surveillance of the disease. It was established in 1987 as a program of the Health Ministry and is coordinated by the Istituto Superiore di Sanità. The early diagnosis performed by the nationwide newborn screening programme, the prompt treatment and the appropriate clinical management of the patients carried out by the Follow-up Centres, and the surveillance of the disease performed by the National Register of infants with congenital hypothyroidism are the components of an integrated approach to the disease which has been successfully established in our country.

The aim of the Register is to monitor efficiency and effectiveness of neonatal screening, to provide disease surveillance and to allow identification of possible aetiological risk factors for the disease. During the past twenty years the active and continuous collaboration between the Register and the Italian Screening and Follow up Centres for Congenital Hypothyroidism allowed to perform a standardization of screening procedures and considerable improvements in the time at starting treatment and in the dose of therapy. Furthermore, the large amount and the high quality of information collected in the Register provided a unique opportunity for research into the disease. This because data collected in the Register are highly representative as referred to the entire Italian population with congenital hypothyroidism. The results derived from the epidemiological studies performed in these years, by using the Register database, contributed to deepen the knowledge of congenital hypothyroidism, to start identifying the most important risk factors for the disease, and to orient molecular studies aimed at identifying new genes involved in the aetiology of this condition.

## Introduction

The main objective of neonatal screening, the eradication of mental retardation after congenital hypothyroidism (CH), has been achieved in all the countries where nationwide screening programmes have been established [1-3]. In Italy the nationwide newborn screening programme for CH began in 1977 as a pilot program in 7 laboratories and then progressively developed all over the country. The 100% coverage of neonatal population has been achieved since the 90's thanks to an efficient network of 26 regional and inter-regional Screening and Follow-up Centres active in our country. At present in all the Italian Screening Centers a biochemical assessment of TSH, and in 11 of the 26 centres also of T4, is performed on dried spot blood within a few days from birth. In all the Centers positive results of screening tests are confirmed by definitive tests of thyroid function on serum. These include TSH, free T4 (FT4) and/or T4. Thyroid ultrasound and/or scintigraphy are generally performed to complete the CH diagnosis. Infants with confirmed primary CH are then referred to the Follow-up Center of their own region for starting replacement therapy. According to international guidelines [1,4,5], when the definitive diagnosis is not established in the neonatal period and a suspicion of transient primary hypothyroidism is present, a reevaluation of diagnosis is performed at the age of 3 years after a withdrawal of the replacement therapy to ascertain the persistence of CH. At that time, serum T4, FT4, and TSH levels are measured, and ultrasound imaging, scintigraphy, and clinical evaluation are performed to establish the definitive diagnosis.

All the Italian Centres in charge of screening, diagnosis and follow-up of infants with CH participate in the Italian National Register of Infants with Congenital Hypothyroidism (INRICH), which performs the nationwide surveillance of the disease. The INRICH was established in 1987 as a program of the Health Ministry [6] and is coordinated by the Istituto Superiore di Sanità. The aim of the INRICH is to monitor efficiency and effectiveness of neonatal screening, to provide disease surveillance and to allow identification of possible aetiological risk factors for CH. Information on new cases with CH are collected in the INRICH by means 3 questionnaires filled in at diagnosis. These include anonymous data concerning CH infants such as screening and confirmatory laboratory tests, information on demographic data, details on clinical state in neonatal period, diagnostic investigations (biochemical determinations, radiography of the knee, thyroid scintigraphy, and ultrasound), information regarding pregnancy, birth, and family background, starting and dose of the replacement therapy. It is important to note that babies with transient hyperthyrotropinemia on the basis of spontaneous normalization of TSH between screening and diagnosis are not recorded in the Register. Since 1991, the INRICH started collecting specific data on the occurrence of congenital anomalies (detected during neonatal period) other than those of the thyroid gland by using a specific reporting form. The Screening Centres are responsible for collecting information in the questionnaires and for the accuracy of their compilation. Data are coded and stored in an informed database at the Istituto Superiore di Sanità and results of the Register are reported in a web site , presented yearly in a national conference, and published in international scientific journals.

## Discussion

During the past twenty years the active and continuous collaboration between the INRICH and the Italian Screening and Follow up Centres allowed to perform a standardization of screening procedures and considerable improvements in the time at starting treatment and in the dose of therapy. In fact, while the median value of infant's age at starting therapy was 23 days between 1987 and 1999, the last analysis of the INRICH data (performed on data referred to babies born between 2000 and 2004) confirmed a reduction of this value (19 days) with significant differences among the 3 diagnoses: agenesia 16 days; ectopia: 15 days; in situ thyroid: 23 days. Similar improvements have been also obtained in dose of L-T4 at starting therapy. The median value of L-T4 dose was 8.0 μg/Kg/day between 1987 and 1999, and 9.6 μg/Kg/day between 2000 and 2004. An analysis on data referring to 2005–2008 is going on and the results are expected further improved.

The INRICH has also allowed to well characterize the Italian population of babies with CH. As expected, in Italy the frequency of the disease is higher in female than in male babies with a F/M sex ratio = 1.7. However, when thyroid disgenesis is considered separately from *in situ *thyroid, the sex ratio results: F/M = 2.0 among babies with thyroid disgenesis and F/M = 1.0 among those with *in situ *thyroid. Moreover, the INRICH data have shown that scintigraphy and/or ultrasonography is performed in the 64% of CH babies before starting therapy and that the different diagnoses are distributed as follows among babies with permanent CH: 40% ectopy, 26% agenesis, 34% in situ thyroid .

The high number of CH infants with confirmed diagnosis recorded in the INRICH (about 3800 at the end of 2007) and the fact that the INRICH is a population-based register have allowed to perform a robust estimation of the CH incidence in our country. This results to be 1:2400 live borns (1995–2003). It is important to note that this estimation is based only on cases with permanent forms of CH. In fact, all the cases with transient hypothyroidism, ascertained by means a re-evaluation of the diagnosis after a withdrawal of the replacement therapy at 3 years of age, are not considered in the incidence estimation. Moreover, to avoid the possibility of including some cases with transient hypothyroidism not re-evaluated yet, only data regarding children older than 3 years at the time of analysis are used. This methodology avoids the danger of drawing conclusions from an overestimation of the CH incidence and allows to ascertain the real impact of this condition on the Italian newborn population. On this regard, the INRICH data have also shown a high frequency of twins in the CH population with a proportion 3-fold higher in the CH population (3.5%) than in the Italian general population (1.1%) [7,8]. For the first time the INRICH data have allowed to estimate the CH incidence in multiple and single deliveries separately. It was found 3-fold higher in multiple (10.1 per 10,000 live births) than in single deliveries (3.2 per 10,000 live births) with a relative risk of CH occurrence in twin deliveries of 3.1 (95% CI, 2.5–3.9) [7]. Moreover, the analysis of re-evaluated infants with high suspicion of transient hypothyroidism recorded in the INRICH has shown a twin prevalence of 1.9% among infants who were affected by permanent CH and 13.2% in those who resulted affected by transient CH. Taken together these findings have demonstrated an increased risk for both permanent and transient CH in multiple than in single deliveries. This increased CH risk in multiple pregnancies has important implications in terms of public health given the high number of induced pregnancies, in Italy as well as in other Western countries, because of the increasing use of techniques of assisted reproduction and drugs inducing ovulation [9,10].

It is important to underline that, beside an efficient nationwide surveillance of the disease, the large amount and the high quality of information collected in the INRICH during these twenty years provided a unique opportunity for research into this condition. This because data collected in the INRICH are highly representative as referred to the entire Italian population of infants with CH. One of the most important results concerns the well known association between congenital malformations and CH [11]. Given the large CH population recorded in the INRICH, it has been possible to study a high number of CH infants with extrathyroidal malformations and compare these data with those of the International Clearinghouse for Birth Defects, the worldwide database collecting information on infants born with congenital malformations [12]. By using this approach, it has been possible to demonstrate that not all congenital extrathyroidal malformations but only anomalies of heart, nervous system, eyes (representing precocious structures in the developing embryo) and multiple congenital malformations are significantly associated to CH [13]. These findings have strongly suggested a very early impairment in the first stages of embryo development with a consequent involvement of different organs and structures. For what specifically concerns cardiac congenital malformations, the comparison of the INRICH data with other national and/or regional registries of congenital malformations included in the database of the International Clearinghouse for Birth Defects, have demonstrated that the most frequent cardiac malformations in the CH population were represented by the atrial septal defects with a rate of 13.8 of 1000 [10]. This finding is different from what found in the general population in which the most frequent cardiac anomalies (3 of 1000) are represented by ventricular septal defects [14]. These peculiar results oriented molecular biologists to focus their investigations on genes involved in both heart and thyroid development. Therefore, as many point mutations have had identified in NKX2.5 trascription factor in families with atrial septal defects [15], the possible involvement of NKX2.5 mutations in thyroid disgenesis was investigated both in vivo model and humans. It was found that mutations in this gene can contribute to thyroid disgenesis phenotype [16].

It has been demonstrated that CH is a multigenic disease [17-20]. However, the occurrence of mutations in genes known to be involved in the development of the disease have been observed only in a small proportion of the CH patients. Moreover, the aetiological role of specific environmental risk factors has not completely elucidated yet. These considerations imply that the etiology of CH is still largely unknown and that further efforts to identify new genetic markers and modifiable (environmental) risk factors are needed to allow an efficient primary prevention of the disease. To this end a population-based case control study was carried out to identify the most important risk factors for permanent and transient forms of CH on the basis of information collected in the INRICH questionnaires [21]. This study showed that many risk factors contribute to the aetiology of CH suggesting a multifactorial origin of the disease in which genetic and environmental (especially iodine deficiency and maternal diabetes) risk factors play a role in the development of the disease. The multifactorial origin of CH was also supported by results obtained in the above mentioned study on CH twins recorded in the INRICH between 1989 and 2000 [7]. This study showed that, despite a low concordance rate (4.3%) for permanent CH observed among twins at birth, a high recurrence risk for the disease was present among siblings of CH cases (Sibling Recurrence Risk = 35.4; 95%CI: 4.7 – 269.3). These findings strongly suggested the occurrence of non-inheritable postzygotic events in the aetiology of CH and that environmental risk factors may act as a trigger on a susceptible genetic background in the aetiology of the disease.

## Conclusion

The surveillance of CH carried out by the INRICH together with the early diagnosis made by the nationwide screening programme, the prompt treatment and the appropriate clinical management of the patients performed by the Italian Follow-up Centres for CH, are elements of an integrated approach to CH which has been successfully established in our country. However, despite the important results obtained in terms of standardization of screening procedures and improvements in time and dose at starting treatment, we are conscious that further efforts have to be made to improve the diagnosis of CH and to make more precocious the therapy establishment in all affected infants. To this end national recommendations on diagnosis and follow-up of CH, resulting from the integration of available guidelines [1,5,22], the Italian Screening and Follow-up Centres experience, and information derived from the INRICH surveillance activity, are needed. These would help to carry out a process of harmonization and optimization of the Italian Screening programme with a consequent guarantee of an optimal quality of life to all CH infants.

For what concerns the INRICH research activity, the results derived from the epidemiological studies performed in these years have contributed to deepen knowledge of CH, to start identifying the most important risk factors for the disease, and to orient molecular biologists towards the identification of new genes involved in the aetiology of the disease. At present, further collaborative research studies based on the INRICH database are going on and our efforts in the field of CH risk factors identification are continuing with the aim of making possible, in a near future, an efficient primary prevention of a disease which still represents the most frequent endocrinopathy in infancy.

Finally, on the basis of our Italian experience we are confident that, as proposed by other Authors [2], the possible availability of a central European wide database on CH would represent a potent tool of surveillance, epidemiological research and knowledge on CH, and that the INRICH could strongly contribute to achieve this goal.

## Competing interests

The authors declare that they have no competing interests.

## Authors' contributions

AO prepared and wrote the manuscript. All the member of the SGCH contributed by giving their data on infants with congenital hypothyroidism to the Italian National Register of Infants with Congenital Hypothyroidism. All the authors read and approved the final manuscript.
